# Reaching activity in parietal area V6A of macaque: eye influence on arm activity or retinocentric coding of reaching movements?

**DOI:** 10.1111/j.1460-9568.2008.06021.x

**Published:** 2008-02

**Authors:** Nicoletta Marzocchi, Rossella Breveglieri, Claudio Galletti, Patrizia Fattori

**Affiliations:** Dipartimento di Fisiologia Umana e Generale, Università di Bologna I-40126 Bologna, Italy

**Keywords:** coordinate transformations, frames of reference, parieto-occipital cortex, reaching execution, reach-to-point

## Abstract

Parietal area V6A contains neurons modulated by the direction of gaze as well as neurons able to code the direction of arm movement. The present study was aimed to disentangle the gaze effect from the effect of reaching activity upon single V6A neurons. To this purpose, we used a visuomotor task in which the direction of arm movement remained constant while the animal changed the direction of gaze. Gaze direction modulated reach-related activity in about two-thirds of tested neurons. In several cases, modulations were not due to the eye-position signal *per se*, the apparent eye-position modulation being just an epiphenomenon. The real modulating factor was the location of reaching target with respect to the point gazed by the animal, that is, the retinotopic coordinates towards which the action of reaching occurred. Comparison of neural discharge of the same cell during execution of foveated and non-foveated reaching movements, performed towards the same or different spatial locations, confirmed that in a part of V6A neurons reaching activity is coded retinocentrically. In other neurons, reaching activity is coded spatially, depending on the direction of reaching movement regardless of where the animal was looking at. The majority of V6A reaching neurons use a system that encompasses both of these reference frames. These results are in line with the view of a progressive visuomotor transformation in the dorsal visual stream, that changes the frame of reference from the retinocentric one, typically used by the visual system, to the arm-centred one, typically used by the motor system.

## Introduction

Area V6A is a cortical visuomotor area located in the caudal part of the superior parietal lobule (see Galletti *et al.*, 1999, 2003). It contains neurons responsive to visual stimuli (Galletti *et al.*, 1996, 1999), as well as cells sensitive to the direction of gaze (Galletti *et al.*, 1995) and cells showing saccade-related activity (Nakamura *et al.*, 1999; Kutz *et al.*, 2003). Area V6A contains also neurons modulated by somatosensory inputs, mainly from the upper limbs (Breveglieri *et al.*, 2002), as well as arm movement-related neurons (Galletti *et al.*, 1997; Fattori *et al.*, 2001).

Recently, it has been reported that V6A reach-related neurons are able to code the direction of arm movement (Fattori *et al.*, 2005). It is worth noting that this reach-related activity was studied with reach-to-point tasks in which the hand was moved towards foveated visual targets, and therefore the different directions of movement of the arm were coupled with different directions of gaze. Given the existence in area V6A of strong gaze modulations (Galletti *et al.*, 1995), it could be that the modulations of reach-related activity in the above recalled tasks actually depended on the effect of the direction of gaze rather than on that of the direction of reaching movement. Although indirect evidence suggested that this was usually not the case (see Fattori *et al.*, 2005), a direct demonstration of separate effects of the direction of gaze and the direction of arm movement on the activity of V6A neurons is still lacking.

In the present study we directly tackled the problem of whether eye-position signals do affect arm movement-related responses in area V6A using a visuomotor task that dissociates the effect of the direction of gaze from that of the direction of arm movement. In this task, the animal reached and touched a target placed in a straight-ahead position while looking at different positions in space. In other words, it performed a constant reach-to-point movement with different directions of gaze. This allowed us to check the effect of eye position on reaching activity regardless of the effect of the direction of arm movement.

We found that reaching activity was modulated by the direction of gaze in a large percentage of V6A neurons. However, we observed that often the eye-position effect affected reaching activity without affecting fixation-related activity. It was evident that in these cases, factors others than the direction of gaze, though depending on it, were responsible for cell modulation. We suggest that one of these factors is the location of reaching target with respect to the location gazed by the animal, that is, the retinotopic location of target. The comparison in the same neuron of reaching activity when the arm movement was executed towards foveated or non-foveated targets confirmed that in at least a part of V6A reaching neurons this was actually the case.

Preliminary results have been presented in abstract form (Marzocchi *et al.*, 2005).

## Materials and methods

### Experimental procedures

Experiments were carried out in accordance with National laws on care and use of laboratory animals and with the European Communities Council Directive of 24 November 1986 (86/609/EEC), and were approved by the Bioethical Committee of the University of Bologna. During training and recording sessions, particular care was taken to prevent behavioural and clinical signs of pain or distress.

Two trained *Macaca fascicularis* of 6 and 4 kg sat in a primate chair and performed a reaching task with their head restrained. Single cell activity was extracellularly recorded from the anterior bank of the parieto-occipital sulcus using glass-coated metal microelectrodes with a tip impedance of 0.8–2 MOhms at 1 kHz. Action potentials were discriminated with a window discriminator (Bak Electronics, Mount Airy, MD, USA). Recording procedures used for one monkey are similar to those reported in Galletti *et al.* (1995). Briefly, spike times were sampled at 1 kHz, eye movements were simultaneously recorded using an infrared oculometer (Dr Bouis, Karlsruhe, Germany) and sampled at 100 Hz. Recording procedures for the second monkey were slightly different and were described in more detail in Kutz *et al.* (2005). Briefly, spikes were sampled at 100 kHz and eye position was simultaneously recorded at 500 Hz. In both cases eye position was controlled by an electronic window (5 × 5 degrees) centred on the fixation target. Behavioural events were recorded with a resolution of 1 ms.

Surgery to implant the recording apparatus was performed in asepsis and under general anaesthesia (sodium thiopenthal, 8 mg/kg/h, i.v.). A full program of postoperative analgesia (ketorolac trometazyn, 1 mg/kg i.m. immediately after surgery, and 1.6 mg/kg i.m. on the following days) and antibiotic care (Ritardomicina®, benzatinic benzylpenicillin + dihydrostreptomycin + streptomycin, 1-1.5 ml/10kg every 5-6 days) followed surgery. Extracellular recording techniques and procedures to reconstruct microelectrode penetrations were similar to those described in other reports (e.g. Galletti *et al.*, 1995, 1996). Area V6A was recognized on functional grounds following the criteria described in Galletti *et al.* (1999), and on cytoarchitectonic criteria according to Luppino *et al.* (2005).

### The constant reaching task

**Fig. 1:**
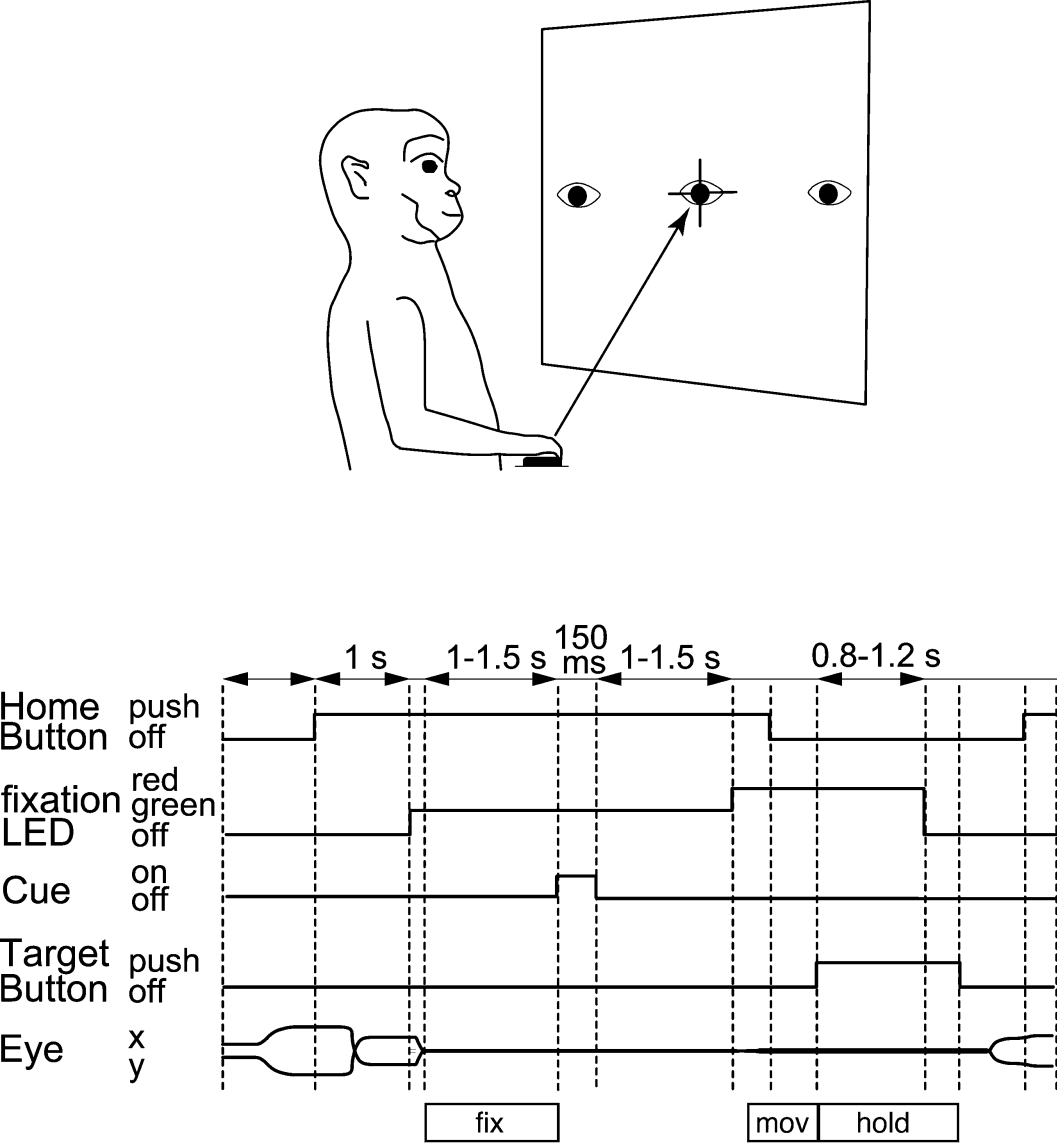
Experimental set-up and time course of the constant reaching task. Top: Scheme of experimental set-up. Reaching movements were performed in darkness, from a home-button (black rectangle) towards a target button (cross) located straight-ahead on a panel in front of the animal. During the execution of the task, the monkey had to fixate a LED on the panel, which could be in one out of three different positions (eye symbols on the panel). Bottom: Time course of the task. From top to bottom: status of the home-button; colour of the fixation target (fixation LED); status of the yellow circular ring indicating the reaching target (cue); status of the target button; examples of eye traces during a single trial (*x* = horizontal component, *y* = vertical component). Lower and upper limits of time intervals are indicated above the scheme. The three labels below the diagram indicate the time epochs that have been analysed (see text).

Data were collected while monkeys were performing a body-out reaching task specifically designed to study the effect of eye position on reach-related neural responses. Reaching target remained always in the same straight-ahead position, whereas fixation point could be in one out of three different positions (Fig. 1, top). Keeping the reaching target constant allowed us to exclude any possible cell modulation related to the direction of arm movement. From here on, this task will be indicated as ‘constant reaching task’. The monkeys performed arm movements with the contralateral limb, with the head restrained, in darkness, and maintaining steady fixation.

As shown in Fig. 1, reaching movements started from a button (home-button, 2.5 cm in diameter) placed outside the animal's field of view, 5 cm in front of the chest, on the mid-sagittal line. Reaching movements transported the hand from the home-button to a target positioned straight-ahead (i.e. at the height of the eyes) on a fronto-parallel panel, which was located 14 cm in front of the animal. The distance between the target button and the home-button was 22 cm. The monkey was required to maintain fixation on the reaching target, or on a different position, 3.7 cm (15.4°) to the right or 3.7 cm to the left of the reaching target. Fixation points were three green/red light-emitting diodes (LEDs; 4 mm in diameter; 1.6° of visual angle) mounted on microswitches embedded in the panel. A circular ring (12 mm in diameter; 4.8° of visual angle), illuminated by a yellow LED, encircled the central fixation target and served as instructional cue for the arm movement and as target of reaching movement.

The time sequence of the reaching task is shown in the bottom part of Fig. 1. A trial began when the monkey decided to press the button near its chest. After pressing the button, the animal was waiting for instructions in complete darkness. It was free to look around and was not required to perform any eye or arm movement. After 1000 ms, one of the three fixation LEDs lit up green. The monkey was required to gaze at the fixation target and to maintain the button pressed while waiting for instructional cue. After a delay of 1000–1500 ms the yellow ring in the central position was lighted up for 150 ms, cueing the target for intervening reaching movement. Then, the monkey had to wait 1000–1500 ms for a change in colour of the fixation LED (green to red) without performing any eye or arm movement. The colour change of fixation target was the go-signal for the monkey to release the home-button and perform an arm movement towards the target button, then to press it. Then, the animal held its hand on the target button till the fixation LED switched off (after 800–1200 ms). The switch off of the fixation LED cued the monkey to release the target button and to press the home-button again. Home-button pressing ended the trial, allowed monkey reward, and started another trial. Fixation had to remain stable on the fixation LED throughout the trial till the fixation LED switched off, otherwise the trial was aborted and a new trial began without any reward.

The correctness of reaching performance was evaluated by a software supervisor system (see Kutz *et al.*, 2005), which checked the status of microswitches (monopolar microswitches, RS components, UK) mounted under the home-button and the LEDs. Button presses/releases were checked with 1 ms resolution.

Fixation of different positions was typically tested as a sequence of randomized triplets in order to collect trials in one position intermingled with the other two. Fifteen trials for each position were collected (45 trials in total).

The reaching task was performed in darkness. The background light was switched on for a few minutes before each block of trials to avoid dark adaptation. To further minimize the role of vision during reaching, the brightness of the fixation LED was reduced, so that it was barely visible during the task.

### The foveal reaching task

Several neurons tested with the constant reaching task underwent also another reaching task, in which the yellow cue indicating the reaching target lit up always in the same position as the fixation target. Therefore, arm-reaching movements were always directed towards foveated targets. The retinotopic coordinates of reaching targets remained constant throughout the task, whereas the direction of movement changed trial by trial according to target position. From here on, this task will be referred to as the ‘foveal reaching task’. As described for the constant reaching task, the foveal reaching task was tested in a sequence of randomized triplets, 15 trials for each condition. Foveal and constant reaching tasks were always presented to the animal in separate blocks.

### The lever task

A small group of neurons was tested with a different visuomotor task, which required the execution of highly stereotyped pull/push movements. Briefly, the animal had to keep its gaze on a fixation spot projected in different positions on a screen placed 57 cm in front of it, and to pull/push a lever placed outside its field of view in response to the onset/change in colour of the fixation spot (for details, see Galletti *et al.*, 1995). Note that in this visuomotor task, as in the constant reaching task, the animal performed a constant arm movement while fixating different spatial locations. This time, however, the arm movement was of small entity (about 1 cm) and was not directed towards a visual target. From here on, this task will be referred to as the ‘lever task’.

### Data analysis

The effect of eye position on neural activity was analysed in different time epochs during the tasks. The time epochs taken into account in our analysis were defined as follows. FIX: steady fixation of the LED during the first delay period (before the yellow cue onset); it was calculated on a single trial basis and therefore varied in duration according to the variable duration of fixation periods. In each trial, the fixation epoch started when the gaze entered the space delimited by the electronic window centred on the fixation point, and ended at the onset of the yellow cue. MOV: from 200 ms before movement onset (home-button release) to movement end (target button pressing). HOLD: from the end of forward reach (target button pressing) to 200 ms before backward movement onset (target button release).

Because the monkey was not required to gaze at the fixation point after the fixation LED was switched off, the eye position was not necessarily maintained still during backward reaching movement from the panel to the home-button. Therefore, we decided not to investigate the eye-position effect on reaching activity during backward arm movements.

Eye-position effect on FIX (fixation-related activity), MOV and HOLD (action epochs) was analysed only in those units that were tested for three fixation targets in at least seven trials for each position. The reasons for this conservative choice are connected to the implicit high variability of biological responses, and are explained in detail in Kutz *et al.* (2003). Significant modulation of neural activity relative to functional epoch or to gaze direction was studied through a two-way analysis of variance (anova) (factor 1: epoch; factor 2: gaze direction). The neural modulation relative to gaze direction was assessed when factor 2 and/or the interaction factor 1 × 2 were significant (*P* < 0.05). Only neurons showing a significant task-related activity were further analysed.

For neurons showing a significant modulation, we statistically compared mean firing rates recorded for different gaze directions during each of the considered functional epochs listed above (one-way anova, *F*-test; significance level: *P* < 0.05).

In neurons showing eye-position modulations both in FIX and in at least one action epoch, *post hoc* tests (Bonferroni-corrected *t*-test, *P* < 0.05) were used to determine specific differences among the gaze directions, therefore obtaining the pattern of modulation for each epoch (i.e. a ranking of gaze directions based on the recorded neural mean activities). We then verified whether the effect of eye position on action epoch(s) was consistent with that on fixation-related activity by comparing the corresponding patterns of modulation.

A *post hoc* test (Bonferroni-corrected *t*-test, *P* < 0.05) was also used to define the preferred condition for each neuron and each epoch.

Population responses of tested neurons were calculated as averaged spike density functions (SDFs). A SDF was calculated (Gaussian kernel, half-width 40 ms) for each neuron included in the analysis, and averaged across all the trials for each tested condition. The peak discharge of the neuron was found in the behavioural epochs of interest, and used to normalize SDF. The normalized SDFs were then averaged to derive population responses.

Whenever possible we compared the strength of neural modulations in the two main tasks we performed (constant reaching and foveal reaching tasks). To this purpose, we defined an index of modulation Im that was calculated separately for each of the two tasks, as follows: 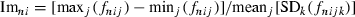 where *f*_*nij*_ is the mean firing frequency of cell *n* during epoch *i* for tested position *j.* Max_*j*_ and min_*j*_ are the functions that take the maximum and the minimum over all positions *j*, respectively. The denominator is an estimate of the spontaneous variability of neural discharge through the trials, calculated as the mean over tested positions of the standard deviation of firing rates recorded in single trials (*k*) of the task. An index near 1 indicates that the spontaneous variability is similar to the putative position-dependent modulation (i.e. no effect). The higher the index, the strongest is the modulation of the neuron in the considered task. Neurons with a modulation index greater than one in both tasks (i.e. neurons modulated in both tasks) were further analysed to identify neurons for which one of the two tasks had a significantly stronger effect. To this purpose, confidence intervals on the modulation indices were estimated using a bootstrap test. *N* firing rates were drawn with replacement from the *N* repetitions of experimentally determined firing rates for each task condition. These synthetic responses were used to recompute the modulation index value. Ten thousand iterations were performed, and confidence intervals were estimated as the range that delimited 95% of the modulation indices (see Batista *et al*., 2007).

All the analyses were performed using custom scripts in Matlab (Mathworks, Natick, MA, USA). All statistical analysis was carried out using the program STATISTICA (StatSoft, Tulsa, OK, USA).

## Results

We recorded the activity of 116 V6A neurons in two animals that performed an arm-reaching task designed to dissociate the effect on neural activity of the direction of gaze from that of the direction of arm movement. The animal was required to reach out a target constantly located in the straight-ahead position while gazing to the target, or to the left or right of it (constant reaching task). Among the 116 cells that underwent this task, 87 fulfilled the requirements to perform the quantitative analysis (see Materials and methods). Forty-three neurons were recorded from one animal and 44 from a second one.

A quantitative evaluation of neural modulations (two-way anova, *P* < 0.05; see Materials and methods) revealed that gaze direction significantly influenced neural activity in the large majority of the neurons studied (84%, 73/87). Only these neurons (from here on called task-related cells) were included in the following analyses, unless differently indicated. The incidence of task-related cells in the two animals was essentially the same.

When considering each behavioural epoch separately, we found that 51% (37/73) of V6A task-related cells showed a fixation-related neural activity (epoch FIX) significantly modulated by eye position (anova, *P* < 0.05). This value is in line with previous studies (Galletti *et al.*, 1995). Most of the task-related V6A cells showed eye-position modulation of neural activity during action epochs. The effect of eye position on neural activity during the execution of arm movement (epoch MOV) and the holding of a static posture of the arm in space (epoch HOLD) will be separately described in the following sections.

### Eye-position effect on cell activity during action epochs

#### Forward arm movement (MOV)

In about 78% (57/73) of task-related V6A neurons the direction of gaze modulated neural activity in epoch MOV. Out of these 57 cells, 28 were modulated by gaze also during the fixation epoch. For these neurons, *post hoc* tests (Bonferroni-corrected *t*-test, *P* < 0.05) were used to determine specific differences among the gaze directions in FIX and MOV, respectively, therefore obtaining the pattern of modulation for each epoch (i.e. a ranking of gaze directions based on the recorded neural mean activities). We found that some neurons showed consistent modulations between FIX and MOV, and some neurons did not (see Table 1). Quite surprisingly, half of the neurons modulated in both FIX and MOV (14/28) showed inconsistent patterns of eye-position modulation during the two epochs.

**Table 1:** Neural modulations of task-related cells in the constant reaching task (*N* = 73)

	Modulated			
		
	Action epoch only	Inconsistent with FIX	Consistent with FIX	Not modulated
MOV	29 (40%)	14 (19%)	14 (19%)	16 (22%)
HOLD	22 (30%)	12 (16%)	18 (25%)	21 (29%)

**Fig. 2:**
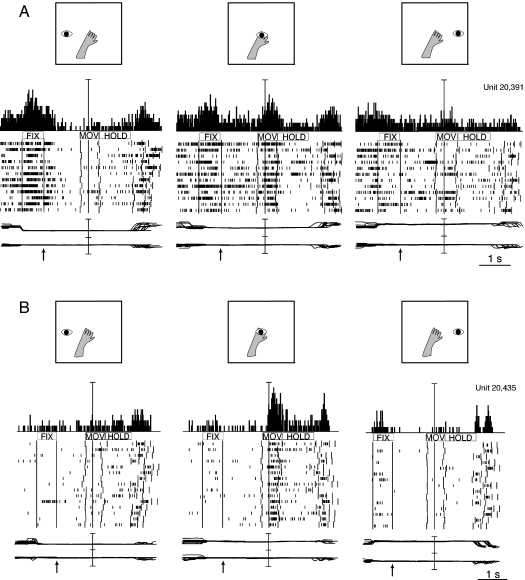
V6A neurons modulated by gaze direction during arm movement execution in the constant reaching task. From top to bottom: sketch of tested conditions (square: panel; eye: fixation point; hand: reached position); peri-event time histogram (PSTH); raster displays of impulse activity; recordings of horizontal (first trace) and vertical (second trace) components of eye positions. Neural activity and eye traces have been aligned twice in each inset: with the onset of target cue (indicated by the arrow) and the onset of reaching movement (indicated by vertical bars on PSTH and eye traces). The mean duration of epochs FIX, MOV and HOLD is indicated below each PSTH. Short vertical ticks in raster displays are spikes. Long vertical ticks among spikes indicate the occurrence of behavioural events. From left to right, the behavioural events are: eye entry in fixation window, target cue onset, go signal, onset and end of reaching movement, end of holding period, end of return reaching movement, trial end. (A) Neuron showing an inconsistent modulation in FIX and in MOV: activity in FIX was higher when the animal looked leftward, in MOV when it looked to the central position. PSTHs: bin width = 15 ms; scale bar = 70 spikes/s. Eye traces: scale bar = 60 degrees. (B) Neuron showing a modulation in epoch MOV during the execution of the constant reaching task, despite the lack of modulation in FIX: the neuron discharged in MOV when the monkey gazed at the centre of the panel and was not activated by the same reaching movement when the animal gazed to the left or to the right. Scale bar in PSTHs: 40 spikes/s. Other details as in (A).

The neuron in Fig. 2A shows an example of this behaviour. During the fixation period, fixation-related activity was higher when the monkey looked leftward and lower when it looked rightward. This pattern of modulation was not consistent with the one observed in epoch MOV, as the activity in MOV was higher when the animal looked straight-ahead than when it looked leftward or rightward. The high activity observed in epoch MOV when the animal looked centrally can not be solely due to an eye-position effect because, if so, MOV activity should be even higher when the animal looked leftward, and this was not the case.

Figure 2A shows another interesting phenomenon. The activity of the neuron dramatically changed after cue onset (yellow LED indicating the location of reaching target), even if the animal was maintaining a steady fixation (see arrows below eye recordings in Fig. 2A), as if the cell was inhibited by the animal preparation of the successive act of reaching. We are aware that the meaning of neural activity after cue presentation is not univocally determined in our task: it could represent an intention-related activity, a premotor activity, a working memory-related activity or even a change in the state of alertness. Specific tasks that dissociate these different aspects are needed to unravel the meaning of this neural discharge. In any case, this abrupt change in neural activity during fixation further demonstrates that the activity of the cell was not only dependent on the direction of gaze.

Twenty-nine out of 73 task-related cells (40% of our cell population) showed evident modulations in MOV without any sign of eye-position effect in FIX (see Table 1). Figure 2B shows an example of this behaviour. When the animal gazed at the central position, the reaching movement evoked a good neural response, whereas MOV response was lacking when the animal looked leftward or rightward (anova, *P* < 0.01). The activity of the cell during FIX was unaffected by eye position (anova, n.s.), thus excluding that MOV modulation was directly dependent on eye-position signal alone.

**Fig. 3:**
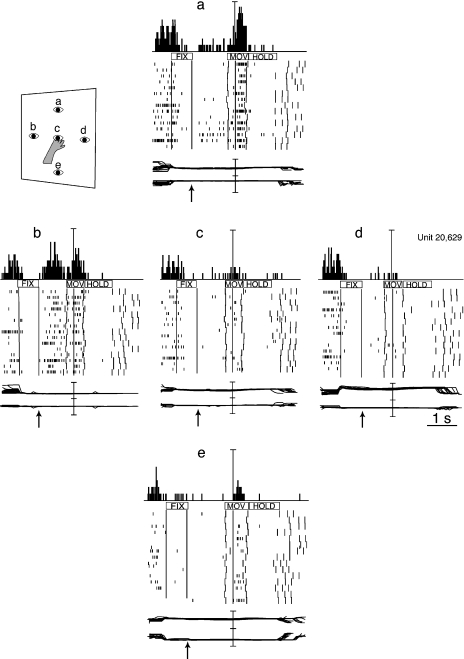
V6A neuron modulated by gaze direction in epoch MOV during the execution of the constant reaching task. Five different directions of gaze were tested (see positions a to e in the upper left inset). Despite the absence of modulations in FIX, the neuron was activated when the fixation point was above or to the left of the reaching target. Scale bar in PSTHs: 35 spikes/s. Other details as in Fig. 2.

The fact that reach-related activity is high when the monkey fixates straight-ahead but low when it performs the same arm movement while looking leftward or rightward, as seen in Fig. 2A and B, could indicate that the cell is activated by the spatial coincidence between target of reaching and fixation point. It is worth noting, however, that not all V6A cells behaved in this way. The cell of Fig. 3, for instance, showed a strong MOV modulation, but the reach-related response was higher when the target of reaching and fixation point were not spatially coincident. We held the neuron of Fig. 3 in record long enough to test it with five fixation points. FIX activity was not modulated, and the cell was almost silent during this epoch regardless of gaze direction. MOV activity was strongly modulated, and the discharge was maximal when the fixation point was above the reached position (Fig. 3a). MOV activity was also high when the fixation point was to the left of the reached position (Fig. 3b), but low for all other fixation point positions, included the central one where fixation point and reaching target positions were the same. In other words, the coincidence between point of fixation and target of reaching was not a compulsory condition to activate V6A cells during action epochs. On the contrary, each cell had its own preferred alignment (or misalignment) between fixation and reaching target that best activated it. As we will see in the following, this is the case also for cells modulated in the HOLD epoch.

The cell of Fig. 3 showed another interesting behaviour. When the animal looked to the left of the reaching target (Fig. 3b) the cell strongly discharged before MOV, right after cue presentation. As previously pointed out, however, the change in neural activity after cue onset may have different explanations that go beyond the scope of this study, and that will be specifically addressed in future experiments.

**Fig. 4:**
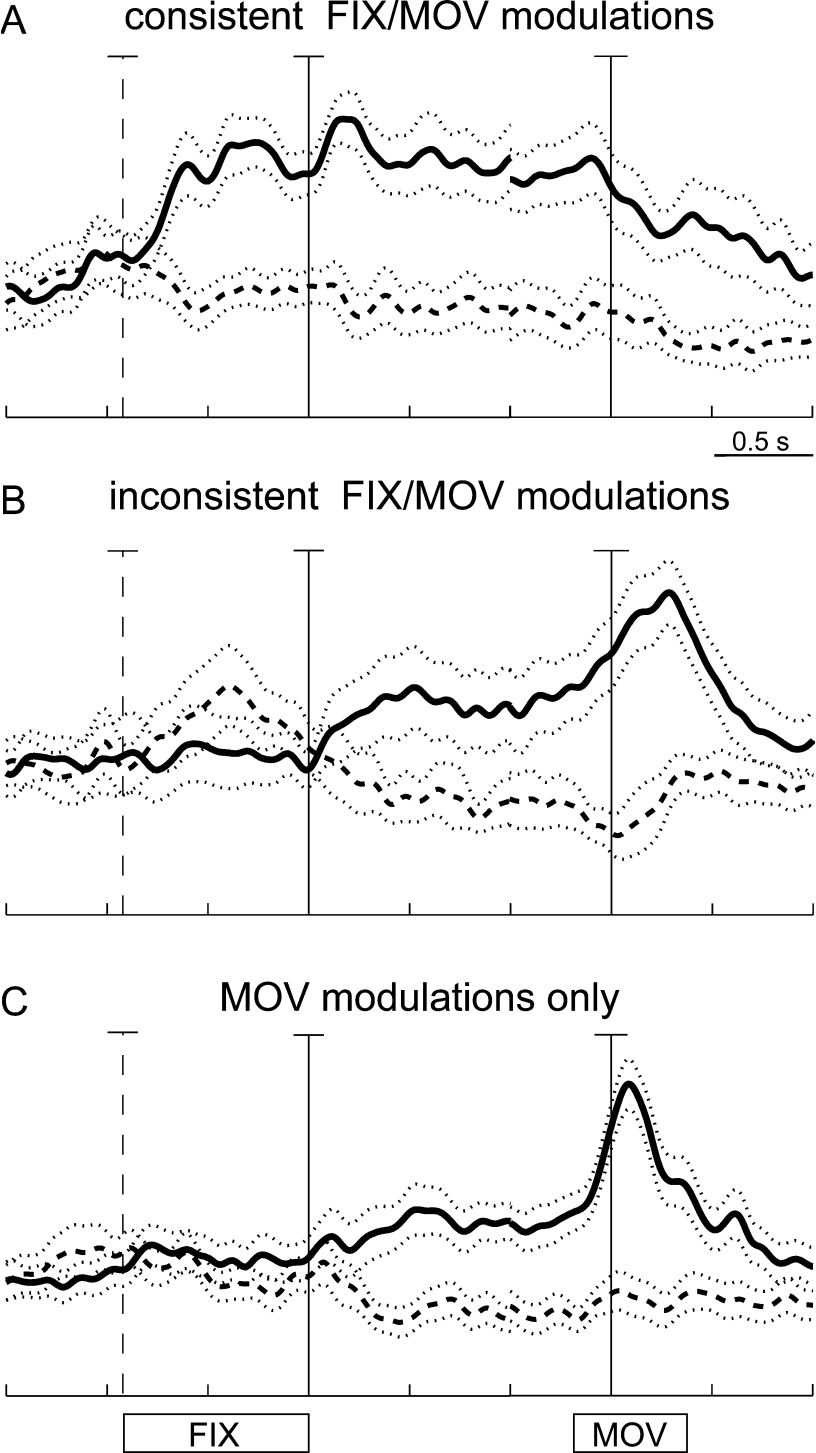
Gaze modulation on the transport phase of reaching (epoch MOV): average population behaviour of V6A neurons modulated in MOV. (A and B) Normalized population responses of V6A neurons showing consistent (A) or inconsistent (B) patterns of modulation in FIX and MOV epochs. (C) Normalized population responses of V6A neurons modulated by gaze direction in MOV but not in FIX epochs. The average SDFs for the preferred (best MOV response) and non-preferred (worst MOV response) conditions are shown as continuous and dashed lines, respectively. Two dotted lines for each SDF indicate the variability band (SEM). The activity of cells in each population was aligned twice (on cue onset and on onset of arm movement, respectively, as indicated by vertical continuous bars). Vertical dashed bar indicates the beginning of FIX. Scale bar in all SDF plots: 0.76.

Figure 4 shows the average behaviour of the V6A task-related cells modulated in MOV epoch. We considered the responses (SDFs) of each neuron in the preferred (continuous curve) and the non-preferred (dashed curve) condition, i.e. the conditions eliciting the best and the worst response, respectively, in MOV epoch. Single neuron responses were normalized and then averaged across the population. Neurons were divided in the three groups, shown in Table 1, based on the type of modulation observed during the fixation interval with respect to MOV epoch: consistent pattern (Bonferroni-corrected *t*-test, *P* < 0.05) in FIX and MOV; inconsistent pattern (Bonferroni-corrected *t*-test, *P* < 0.05) in FIX and MOV; no eye-position effect in FIX.

Figure 4A shows the average responses of V6A neurons with consistent pattern of modulation in FIX and MOV. The mean activity diverged at the beginning of fixation, according to the preferred direction of gaze. In the preferred condition (continuous line) the neural activity remained high after the onset of cue signal. On the contrary, it stayed at baseline level when the task was performed with the non-preferred gaze direction. In other words, neural activity was dominated by the gaze signal, no matter whether or not the animal was performing arm-reaching movements.

Figure 4B shows the average responses of neurons with inconsistent patterns of modulation in epochs FIX and MOV. During the first part of fixation, the activity in the non-preferred condition (for reaching) was slightly higher with respect to that in the preferred condition. The activity began to significantly change around the time of cue signal, then changed dramatically after it, progressively increasing in the preferred condition and decreasing in the non-preferred condition, achieving a maximum and a minimum, respectively, during MOV epoch. Note that this type of cell is modulated by gaze and arm movement in an opposite way.

Figure 4C shows the average responses of neurons in which the gaze signal significantly modulates arm-reaching activity but not fixation activity. Population activity progressively increased in the preferred condition after cue signal, peaking at MOV epoch, and then decreased during HOLD period, whereas in the non-preferred condition the activity remained quite constant below the level observed during FIX.

#### Holding phase (HOLD)

**Fig. 5:**
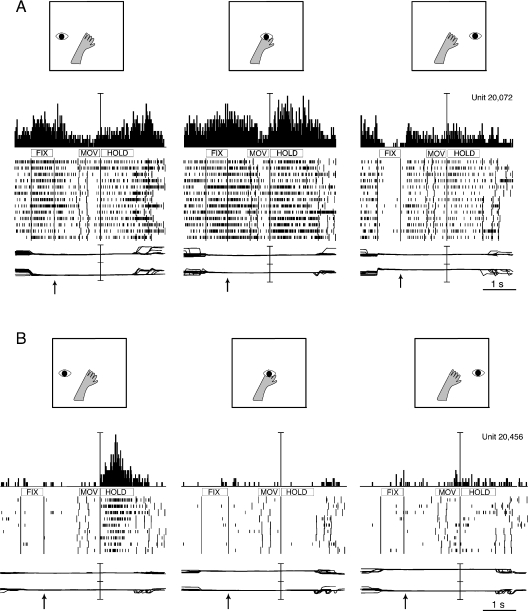
V6A neurons modulated by gaze direction in epoch HOLD during the execution of the constant reaching task. Neural activity and eye traces have been aligned twice in each inset: with the onset of target cue (indicated by the arrow) and the beginning of holding phase. (A) Neuron modulated in FIX and HOLD, showing inconsistent patterns of modulation, demonstrating that eye-position signals *per se* were not responsible for the observed behaviour in HOLD. Scale bar in PSTHs: 70 spikes/s. (B) Neuron modulated in epoch HOLD. The neuron discharged only during holding of the hand on the right of fixation point. Note that this cell does not discharge at all during hand holding in the same spatial position when the gaze is directed centrally or to the right. Scale bar in PSTHs: 75 spikes/s. Other details as in Fig. 2.

As summarized in Table 1, we found that in 71% of task-related neurons (52/73) the activity in HOLD was significantly modulated by the direction of gaze; 30 of these neurons were also modulated during FIX, the remaining 22 were not. More than one-third of the cells modulated in HOLD and FIX (12/30) showed inconsistent modulations in the two epochs. One of these neurons is shown in Fig. 5A. The cell was almost completely inhibited during FIX when the animal looked rightward, whereas it strongly discharged during this epoch when the animal looked at the central position or leftward. During HOLD epoch, the neuron showed the strongest activation when the monkey fixated the central position, whereas activity was weaker when looking leftward or rightward. Note that when the animal looked leftward the activity in FIX was about the same as when it looked centrally, but the HOLD activity was much lower looking leftward than looking centrally. In other words, eye position alone was not the critical factor modulating the action epoch.

Similar to the examples in Figs 2 and 3, also the cell shown in Fig. 5A changed abruptly its activity after the presentation of instructional cue. In particular, when the animal directed its gaze rightward, the cell became almost silent; then, after cue onset, neural activity went up soon, achieving a level similar to the one the cell had before fixation. In other words, rightward fixations strongly inhibited the cell, but this effect disappeared as soon as the instructional cue appeared, even if the animal maintained a steady fixation (see arrow below eye recordings). Exactly the opposite happened when the animal looked leftward: the activity suddenly increased at the beginning of fixation and then decreased after cue onset, achieving also in this case a level comparable to the one the cell had before fixation. As already pointed out for the cell of Fig. 2A, the change in neural activity after cue onset may have different explanations, that go beyond the scope of this study.

In 22 out of 73 task-related cells there was a significant modulation of HOLD activity without a concurrent significant eye-position modulation of FIX activity. Figure 5B shows one of these cells. It was almost silent except when the animal was holding its hand on the reaching target while looking to the left of it (left part of Fig. 5B). Certainly, eye-position signal *per se* is not the critical factor modulating the action epoch (HOLD) of this cell.

**Fig. 6:**
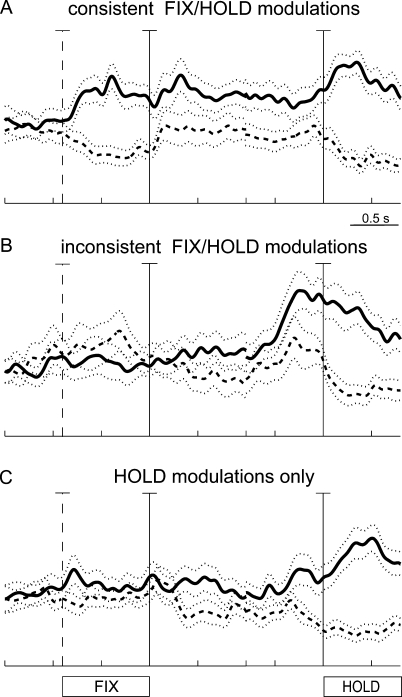
Gaze modulation on the holding phase of reaching (epoch HOLD): average population behaviour of V6A neurons modulated in HOLD. (A and B) Normalized population responses of V6A neurons showing consistent (A) or inconsistent (B) patterns of modulation in FIX and HOLD epochs. (C) Normalized population responses of V6A neurons modulated by gaze direction in HOLD but not in FIX epochs. Preferred/non-preferred conditions were, respectively, the one with the highest activity in HOLD epoch, and the one with the lowest. The activity of cells in each population was aligned twice (on cue onset and on onset of hold epoch, respectively). Other details as in Fig. 4.

The same population analysis previously described for neurons modulated in the MOV epoch (see Fig. 4) was repeated for neurons modulated in HOLD, as shown in Fig. 6. It is important to note that the subpopulation of neurons included in this analysis was partially different from the one considered in Fig. 4. For example, the neuron in Fig. 5, which was modulated during HOLD but not during MOV, was included only in the analysis of Fig. 6. Neurons modulated in HOLD were divided in three groups shown in Fig. 6, based on the relationships between gaze and HOLD modulations (see Materials and methods, *post hoc* tests). For each neuron we took into account the response in the preferred condition (condition with the highest activity in HOLD epoch) and in the non-preferred condition (worst response in HOLD). Continuous lines represent the preferred condition, and the dashed lines the non-preferred one. Population responses for neurons modulated in HOLD presented similar features to the ones observed for neurons modulated in MOV (compare Fig. 6 with Fig. 4). Preferred and non-preferred responses in HOLD were well discriminated in all three groups of neurons, indicating a good range of modulation in all types of cell. For neurons showing patterns of modulation of gaze and action epoch, which were consistent one to another (Fig. 6A), the separation between the two curves started with the beginning of fixation, as expected, and was maintained all throughout the duration of the task, suggesting that for these neurons the modulation was mainly driven by the gaze signal.

For the other two groups, the separation of the two curves took place about 200 ms after cue presentation. The curves remained quite separated before action epochs and diverged significantly during action epochs, in particular during HOLD.

As repeatedly recalled above, the reaching target in constant reaching task occupied a fixed position in space, so the direction of reaching movement was constant. However, because of different gaze directions, the arm movement was directed toward different locations in eye-centred coordinates (different retinotopic locations of the target). When the animal looked at the central fixation point, the reaching movement was directed toward the point of fixation, i.e. the animal executed a foveal reaching. On the contrary, when the animal looked leftward or rightward the reaching movement was toward peripheral retinal locations. Similarly, during HOLD phase, the hand occupied foveal or peripheral positions, respectively, according to the direction of gaze.

Our data show that V6A reaching cells can be activated either when the reaching target is spatially coincident with the fixation point (Figs 2A and B, and 5A) or when they are not (Figs 3 and 5B). Note that in this latter case, it is not the fact that reaching and fixation targets are spatially separated that activates the cells, but the fact that the reaching point is located on a specific side with respect to the fixation point (e.g. to the right of the fixation point). In other words, the cell is selectively responsive to a specific retinotopic location of reaching target.

**Fig. 7:**
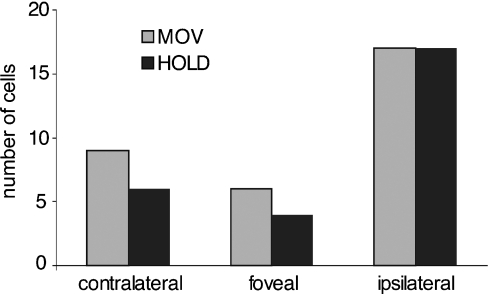
Retinal locations of reaching target preferred by V6A cells during arm movement (grey) and hand holding (black) in constant reaching tasks. The histogram reports the number of neurons modulated in a given action epoch preferring reaching movements executed in the contralateral or ipsilateral visual fields with respect to the recording side. ‘Foveal’ indicates cells preferring reaching targets spatially coincident with the direction of gaze. For each epoch, only neurons with a statistically significant preferred position (Bonferroni-corrected *t*-test, *P* < 0.05) were included in the distribution.

Figure 7 summarizes the distribution of preferred retinal locations of reaching target in our V6A cell population. As we recorded data in both hemispheres, the distribution of peripheral preferred positions is represented in the figure in terms of ipsi- and contralateral hemifield. It is important to note that the preferred position was defined as ipsi- or contralateral with respect to the recording side, and not with respect to the reaching arm. Neurons were included in the distribution only if their activity in the preferred position was statistically different (Bonferroni-corrected *t*-test, *P* < 0.05) with respect to the others. Figure 7 shows the preferred locations for both epochs MOV and HOLD. The distribution of preferred locations was biased towards ipsilateral positions in both cases (chi-squared test, *P* < 0.05), in other words many V6A neurons showed the strongest activity during reaching towards a target located in the visual hemifield ipsilateral with respect to the recording hemisphere.

### Eye-position effect in other visuomotor tasks

**Fig. 8:**
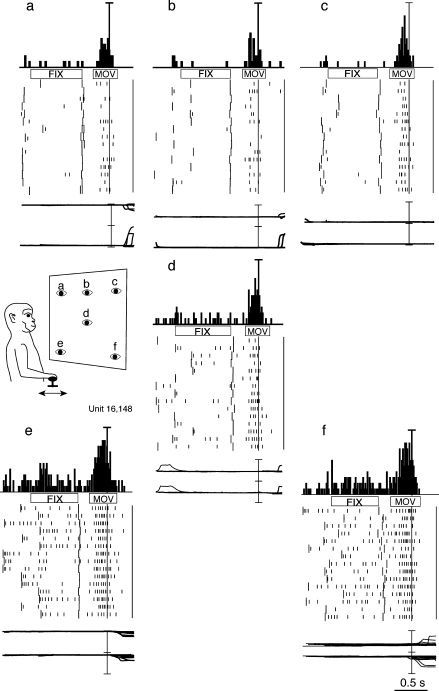
Tuning of arm movement-related activity of a V6A neuron during the execution of the lever task. Sequence and type of data in each inset are as in Fig. 2. Insets are positioned according to the location gazed by the monkey on the panel it faced (see letters a to f in the sketch on the left side of the figure). In each inset, neural activity and eye traces have been aligned on the onset of arm movement (lever-push). Behavioural events (long ticks in raster displays) from left to right are: trial begin; go signal for the arm movement; arm-movement onset; trial end. FIX epoch: from beginning of fixation to go-signal; MOV epoch: push movement of the lever. The arm movement was performed in darkness outside the field of view, while the monkey was fixating in different directions. Both fixation-related (FIX) and movement-related (MOV) activities increased when the animal looked downward. Scale bar in PSTHs: 50 spikes/s.

A small group of neurons (*N* = 21) underwent a simple visuomotor task (lever task) in which the animal was required to perform stereotyped arm movements while maintaining steady fixation on different spatial locations. Five out of 21 neurons showed arm movement-related activations modulated by the direction of gaze. One of these neurons is illustrated in Fig. 8. The fixation-related activity of the cell clearly depended on the direction of gaze, being higher for downward directions of gaze. The cell also showed a clear discharge when the monkey pushed the lever no matter where it was looking at. The discharge began about 200 ms before the arm movement and stopped just after the onset of movement. This movement-related activity was clearly modulated by eye position, being higher when the monkey fixated downward (Fig. 8e and f). The patterns of modulation of fixation-related and movement-related activities were consistent, both preferring downwards directions of gaze. The behaviour of this cell demonstrates that an effect of eye position on the arm movement-related activity can be present also when the movement of the arm is not directed towards a visual target and is completely performed outside the visual field.

Most of the neurons that underwent the constant reaching task (54/87) were also tested in the foveal reaching task. In this task the animals reached foveated targets located in different positions on the panel they faced, performing reaching movements in different directions according to the target location.

**Fig. 9:**
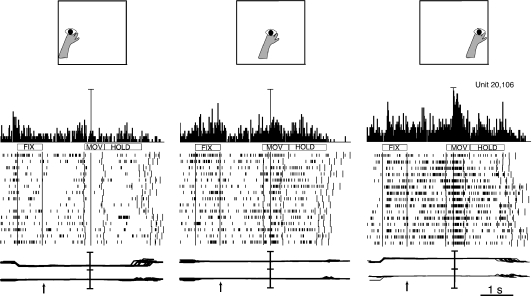
V6A neuron spatially tuned during the execution of reaching movements in the foveal reaching task. Note that in this task the location of reaching target and that of fixation point were always spatially coincident. Scale bar in PSTHs: 85 spikes/s. Other details as in Fig. 2.

Figure 9 shows an example of a cell modulated in the foveal reaching task, both in FIX and MOV in a consistent way. When the animal gazed and reached the target in the left part of the panel, cell activity was low both in FIX and MOV epochs. When the monkey gazed and reached out the target in the central part of the panel or rightwards, both FIX and MOV activities were higher. We found that about half of tested neurons were sensitive to the direction of movement or the position of arm in space, in agreement with a previous report (Fattori *et al.*, 2005). In particular, 31 out of 54 neurons (57%) showed a significant modulation in MOV, and 31 out of 54 (57%) in HOLD (anova, *P* < 0.05).

**Fig. 10:**
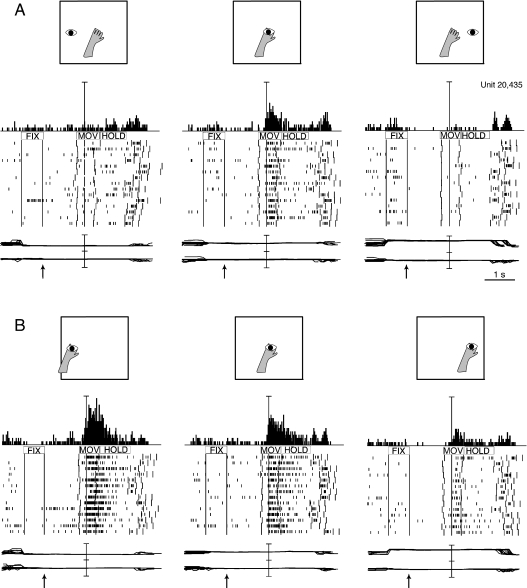
V6A neuron modulated in epoch MOV, both in constant reaching (A) and in foveal reaching (B) tasks. In the upper part of each inset, a sketch of the panel indicates the positions of fixation (eye) and reaching (hand) targets. The neuron was activated only by reaching movements toward a target foveated by the animal (A, centre); this discharge is modulated by the direction of reaching movement (B). Scale bar in PSTHs: 65 spikes/s. Other details as in Fig. 2.

For the 54 cells that underwent both constant reaching and foveal reaching tasks, we tested the same three positions along a horizontal line, e.g. the central one, 15.4 ° to the left and 15.4 ° to the right. Figure 10 shows the responses of the same cell as in Fig. 2B during the constant reaching task (Fig. 10A) and foveal reaching task (Fig. 10B). Figure 10A shows the effect of direction of gaze on reaching activity, and Fig. 10B the effect of direction of arm movement plus that of direction of gaze on reaching activity. Note that the cell discharged during arm movement epoch only when the reach was directed toward the fixation point (Fig. 10A, centre). When foveal reaching movements were performed towards different spatial positions (Fig. 10B), the neuron showed MOV activations in all cases, but the amplitude of response changed according to the direction of reaching (and the direction of gaze) (anova, *P* < 0.01). In particular, the best response was evoked for leftward reaching (with the animal looking leftward too). When considering the response of this cell in the two conditions with leftward fixation (left parts of Fig. 10A and B), the activities recorded during arm movement were deeply different, demonstrating that the parameter driving neural discharge during MOV was not the position of the eye.

The same conclusion is achieved if one considers that the FIX activity in both tasks was completely unaffected by the direction of gaze. The coincidence of reaching target with fixation point was necessary to activate the cell during MOV. When this condition was verified, a gain effect could be observed, likely dependent on the direction of movement of the arm (leftward reaching preferred).

**Fig. 11:**
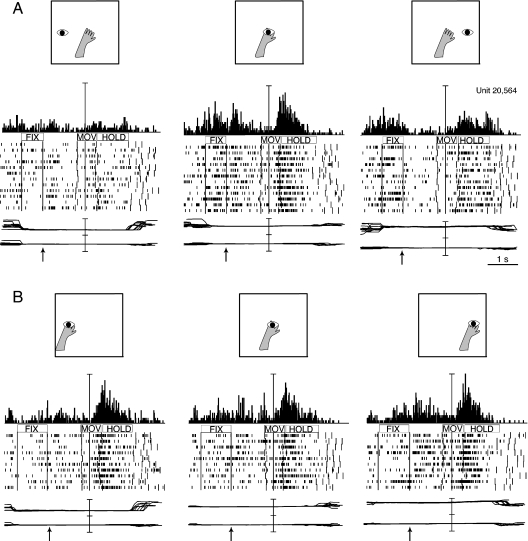
V6A neuron modulated in epoch HOLD, in the constant reaching task (A), but not in the foveal reaching task (B) The neuron was activated by holding of the hand on the gazed position (A, centre) and this activity is not modulated by the direction of reaching movement (B). Scale bar in PSTHs: 100 spikes/s. Other details as in Figs 2 and 10.

A different neural behaviour is shown in Fig. 11. The neuron was activated during the holding phase in the constant reaching task (anova, *P* < 0.01), showing the strongest discharge when the hand was held on the position gazed by the animal (Fig. 11A, centre). In the foveal reaching task (Fig. 11B), the discharge of the same cell during HOLD was not significantly different in the three tested positions (anova, n.s.). In other words, the intensity of HOLD discharge was not dependent on the position in space of the hand during HOLD, but on the position of the hand with respect to the fixation point, i.e. on the retinotopic location of the hand.

When a cell showed an action epoch that was strongly modulated in the constant reaching task but not in the foveal reaching task (as the cell shown in Fig. 11), we can argue that that cell is able to encode the retinotopic location of the target towards which the hand is directed (or where it stands with respect to where the animal is looking at) but not its spatial location in either head-, arm- or body-centred coordinates. Conversely, when a cell showed an action epoch that was modulated in the foveal reaching task but not in the constant reaching task, we can argue that this cell is able to encode the spatial direction/location of hand movement/position, but not its retinotopic counterpart.

**Fig. 12:**
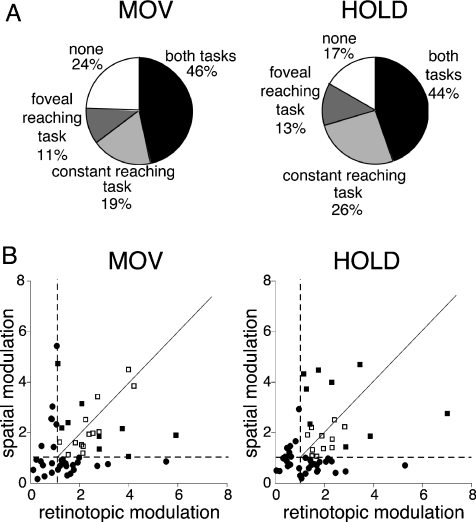
Behaviour of V6A cell population tested in both constant reaching and foveal reaching tasks (*N* = 54). (A) Pie diagrams representing the incidence of neurons modulated in MOV and HOLD in the constant and foveal reaching tasks. (B) Distribution of modulation indices (Im) calculated for MOV (left) and HOLD (right) in the constant reaching task (retinotopic modulation) and in the foveal reaching task (spatial modulation). Each point represents one neuron. Dashed lines drawn at Im level = 1 represent the significance levels, as an Im value ≤ 1 represents a modulation in the range of spontaneous variability. Filled circles indicate neurons with a retinocentric or spatial frame of reference (points with just one value > 1), or neurons not modulated by either tasks. Filled squares indicate neurons with a mixed retinocentric/spatial frame of reference whose bootstrap-estimated confidence intervals do not cross the unity diagonal. Empty squares indicate neurons modulated with a similar strength by both tasks.

Figure 12A shows the incidence of the different types of modulation in our V6A neuronal population. Though neurons with any type of modulation were found, the most relevant behaviours are represented by neurons that are modulated in both tasks, as the cell in Fig. 10 (about 45% for both epochs), that is neurons whose main job is likely to encode reaching phases in a mixed frame of reference that encompasses information both about the relative position of the reaching target with respect to gaze direction and its spatial position. A portion of V6A neurons is modulated in the constant reaching task only (19% and 26% in MOV and HOLD, respectively), as the cell in Fig. 11, that is they are neurons sensitive to the retinotopic position of the target but not to its spatial position. The cells modulated only in the foveal reaching task, i.e. cells encoding only the spatial position of the target during reaching phases, represent a minority (11% and 13%) of our neuronal population.

We calculated a modulation index (see Materials and methods) that measured the efficacy of the task in modulating V6A neural activity during arm movement or holding phase. Data obtained from our V6A population are summarized in Fig. 12B. Each point in the two plots of the figure represents one neuron. The *X*-value of each point is the value of the modulation index calculated for that neuron in the constant reaching task (i.e. the value of its sensitivity to the retinotopic position of the target), the *Y*-value is calculated in the foveal reaching task (i.e. the value of its spatial modulation). Filled circles with both *X*- and *Y*-values ≤ 1 represent cells not significantly modulated by either tasks. Filled circles with just one value > 1 represent cells significantly modulated by only one of the two tasks. Squares with both *X*- and *Y*-values > 1, represent neurons significantly modulated by, both tasks. In agreement with the results shown in Fig. 12A, Fig. 12B shows that the majority of V6A cells encode reaching action in a mixed retinocentric/spatial frame of reference, while a minority encode reaching action in a retinocentric or spatial frame of reference. For neurons showing a mixed coding of reaching action, we used a bootstrap test to verify what parameter (spatial vs retinotopic) was affecting more strongly the cells responses. Filled squares in Fig. 12B indicate neurons with a mixed retinocentric/spatial frame of reference whose bootstrap-estimated confidence intervals do not cross the unity diagonal. For these neurons, we can assert with confidence that the neuron is more sensitive to retinotopic (below the diagonal) or spatial (above the diagonal) modulation. For both MOV and HOLD epochs a consistent group of neurons (empty squares) were modulated with a similar strength by both tasks.

## Discussion

### Eye-position effect

We recorded neuronal activity in the parietal area V6A of two monkeys that performed a constant reaching movement towards a target in front of them while gazing at different positions. We found that gaze direction modulates neural activity in a large majority of V6A neurons, in terms of main effects and interaction between eye position and task epoch. In particular, gaze direction modulates the fixation-related activity of about 50% of tested cells, in good agreement with what we found in a previous study (Galletti *et al.*, 1995). Gaze direction also modulates neural activity during reaching movement and/or during holding of a static posture of the arm in space in about two-thirds of tested neurons.

Because we know that V6A is a visual area (Galletti *et al.*, 1999), it was important to exclude that observed neural modulations were the result of visual stimulation. For this reason, we performed the experiments in darkness, using a very dim and small fixation target. We also placed the home-button outside the field of view of the animal so to exclude that arm movement-related responses, which usually began at home-button release or earlier, were due to a visual stimulation produced by the arm sweeping across the visual field.

We also know that V6A visual neurons mainly represent the contralateral part of the visual field (Galletti *et al.*, 1999). Thus, if modulations during action epochs were dependent on a stimulation of the cell visual receptive field by the arm in motion, we would have expected to find a large majority of cells showing stronger activity when the reaching target was in the contralateral visual hemifield with respect to the recording side. On the contrary, we found that ipsilateral positions were more represented than contralateral ones (see Fig. 7).

In conclusion, observed modulations were not the result of visual stimulations. They were actually due to an interaction between direction of gaze and execution of a reaching movement.

### Possible functional significance of the gaze modulation of reaching activity

Our results show that in some cases eye position modulates only fixation-related activity, and in other cases it modulates only reaching activity. Often, the gaze effect acts on both fixation-related and reaching activities. In these cases, we often observed a different spatial pattern of modulation of gaze effect so that, for instance, fixation-related activity increased when the animal looked leftward but reaching activity increased when it looked rightward, and vice versa. It is therefore evident that eye-position signal has not a simple, monotonic effect on the cell reaching activity. In other words, the gaze effect can not explain the modulation that occurs in action epochs according to the direction of gaze. It could be that these neurons are encoding the position of the target of reaching with respect to the gaze direction, i.e. its retinotopic position. In other words, it could be that the neural discharge of these neurons during action epochs encodes whether the hand is directed towards (or is standing on) the point the animal is looking at, or towards (or standing on) another, non-foveated location. Along this line of thinking, we could suppose that in V6A there are cells discharging strongly for reaching actions directed towards the point gazed by the animal, and other cells preferring actions directed away from the point gazed by the monkey, leftward, rightward, upward or downward with respect to it. This is in line with what we have found in this work.

Some of the V6A neurons, like the cell shown in Fig. 11, show modulations that are dependent on both retinotopic (eye-centred) end-position of reaching movement (or retinotopic position of hand at the end of reaching movement) and the direction of hand movement, or the position of the hand in peripersonal space, in body (head? arm?)-centred coordinates. Some other neurons, like that shown in Fig. 10, are sensitive only to the retinotopic (eye-centred) end-position of reaching movement (or the retinotopic hand position at the end of reaching movement).

Less represented in our sample are the cells modulated only by the direction of movement, or the position in space of the hand.

The existence of a spatial tuning of V6A neural activity during reaching actions had been already demonstrated in a previous work (Fattori *et al.*, 2005). It is clear from the present data that the coding of target spatial coordinates is only a component of a more complex coordinate system, that involves both retinotopic and body-centred frames. We do not know from the present data how the spatial reaching selectivity is differently affected by the gaze direction. To answer this question and to conclusively understand the frame (or frames) of reference used by V6A neurons to code reaching actions, a more complete set of experimental conditions is required. The comparison between the two tasks presented in this paper gave us some suggestions about the functional significance of the observed modulations, which will be further investigated by future studies.

It should also be noted that in our tasks the direction of movement always covariates with the spatial location of reaching target, as the initial hand position was always the same. Therefore, it could be that a cell modulated by the direction of movement is actually encoding the spatial location of reaching target in a body (arm?, head?)-centred frame of reference. Specific tasks dissociating the direction of movement of the arm from the spatial location of reaching target are needed to verify this point.

We could expect that for some of the neurons the lack of modulation might simply reflect the fact that the cell was tuned for an untested direction (most of the cells were tested along the horizontal axis). According to this view, the number of neurons modulated in either task could be underestimated. Testing along more directions should only issue in a scaling up of modulated fractions in each one of the tasks, and therefore in a higher number of neurons showing a mixed coding of eye-centred and spatial coordinates.

The low percentage of neurons modulated only by the direction of reaching or by the hand position in space suggests that encoding arm movement or position *per se* is likely not the main job of V6A. Area V6A is rich in visual cells able to encode the visual space and contains also cells able to encode the object location in spatial (non-retinocentric) coordinates (Galletti *et al.*, 1993, 1995, 2003; Galletti & Fattori, 2002). Therefore, V6A could be involved in early coordinate transformations from retinal (eye-centred) to spatial (arm-centred?), and this could explain the presence within the area, together with reaching cells organized in retinotopic or spatial frames of reference, of a consistent number of cells organized in a mixed retinocentric/spatial frame of reference (see Fig. 12).

The direct connection of V6A with dorsal premotor cortex (PMd; Matelli *et al.*, 1998; Shipp *et al.*, 1998; Galletti *et al.*, 2001) and the prevailing arm-centred coding of reaching activity in the PMd (Caminiti *et al.*, 1991; Cisek & Kalaska, 2002) support the view of progressive visuomotor transformations in the dorsal visual stream changing the frame of reference from the retinocentric one to the arm-centred one.

### Comparison with other studies

It has been repeatedly reported that eye position is effective in modulating reach-related activity in many cortical and subcortical areas involved in planning and preparation of visually guided arm movements. Together with many works exploring the role of eye position on the neuronal activity during the delay before reaching movements (Batista *et al.*, 1999, 2007; Cisek & Kalaska, 2002; Pesaran *et al.*, 2006), only a few works have investigated this issue during movement epochs in reaching tasks. A gaze influence on neural responses during reaching was demonstrated in superior colliculus (Stuphorn *et al.*, 2000), cortical areas 5 (Buneo *et al.*, 2002), 7m (Ferraina *et al.*, 1997) and area PEc (Battaglia-Mayer *et al.*, 2000, 2001; Ferraina *et al.*, 2001), as well as in ventral premotor cortex (Mushiake *et al.*, 1997) and PMd (Boussaoud *et al.*, 1998).

In the study of Boussaoud and colleagues (Boussaoud *et al.*, 1998), the animal performed stereotyped limb movements outside the field of view. The direction of gaze was effective in modulating reaching activity of a large part of tested neurons, similarly to what we have observed in V6A using the lever task.

The other mentioned studies regarding the superior parietal lobule used a centre-out reaching task, in which the hand, standing at the centre of a panel facing the animal, was displaced laterally towards peripheral target positions. Despite the difference with our three-dimensional reaching tasks, gaze modulation of reaching activity was similar to that we find here in V6A.

Stuphorn and colleagues (Stuphorn *et al.*, 2000) used a reaching task in which the hand started the movement near the body and reached a target in the peripersonal space, as in our tasks. They found a large group of neurons in the superior colliculus discharging only when the monkey reached for targets having specific coordinates in relation to gaze axis, either in contralateral or ipsilateral hemifield, similarly to what we find here for V6A.

As far as the frame of reference for reaching is concerned, Batista and colleagues (Batista *et al.*, 1999) were the first who investigated the problem in the posterior parietal cortex. They demonstrated that reaching movements in the medial intraparietal area are planned in eye-centred coordinates. The same question was addressed by two recent works, investigating the frames of reference used by neurons in the PMd during movement planning (Pesaran *et al.*, 2006; Batista *et al.*, 2007). Both studies demonstrated that PMd neurons are influenced by both the eye- and limb-centred locations of reaching targets. Present evidence extends the notion of a retinocentric code for reaching from planning to action epochs (execution of reaching and final position of hand in space). According to the present data, the majority of V6A reaching neurons encode action epochs in a mixed retinocentric/spatial frame of reference. These results are in line with the view that V6A is a node of the medio-dorsal visual stream, which is involved in the online control of reach-to-grasp movements (Galletti *et al.*, 2003). To do its job, V6A needs to transform visual information, typically encoded in a retinocentric frame of reference, in the arm-centred or body-centred frame of reference used by the motor system. The presence in V6A of both reference frames fully agrees with this view.

## Abbreviations

LED: light-emitting diode
PMd: dorsal premotor cortex
PSTH: peri-event time histogram
SDF: spike density function
V6A: area V6A
